# Redefining the health system: A proposed updated framework of a systems approach to health

**DOI:** 10.3389/fpubh.2022.956487

**Published:** 2022-08-15

**Authors:** Farshid Alaeddini, Hamed Tavolinejad, Hamid Esmailzadeh

**Affiliations:** ^1^Tehran Heart Center, Cardiovascular Diseases Research Institute, Tehran University of Medical Sciences, Tehran, Iran; ^2^Health Information Management Research Center, Tehran University of Medical Sciences, Tehran, Iran

**Keywords:** health system, systems thinking, health system actors, support system, health system - organization and administration

## Abstract

Defining the health system, as a multidimensional and complex structure, is challenging, and the existing definitions often fail to incorporate the various levels and functions involved in a single system definition. An ideal framework should be easy to evaluate, allow for comparison, and be divisible into smaller sub-systems for easier interpretation. This paper concisely explores a novel framework to perceive health systems. As in any system, it is important to accurately define the health system's input, process, and output, as the cornerstone of evaluating any system is to assess outputs with regard to inputs besides analyzing outcomes, impact, objectives, and values. Since the raison d'être of the health system is to improve health in society, it is proposed that the input can be considered as the population subject to the system's process, and the output as the population with improved health status. This paper also proposes defining support systems, whose input and output are needs and parts of the process in the main system, respectively. Example support systems include the health evidence production or education and development of human resources systems. Instead of considering all functions as part of the main system, this concept allows implementation and assessment of policies in various levels of health systems to be simplified, as each support system can be separately evaluated with clear functions.

## Introduction

Health systems around the world play a vital role in shaping the health outcomes of individuals and societies (1). Their impact even extends beyond this point, as health is established as an important determinant of sustainable economic growth, security, equity, and effective governance (2, 3). Health systems are complex and multi-dimensional structures operating as dynamic social systems, for which various definitions have been proposed (1, 4–8). However, the existing definitions of health systems are nebulous and often reductionist (2). Furthermore, current definitions are disparate and do not enable comparison between countries (9, 10).

Perhaps the most extensive description of a health system so far has been provided by the WHO. According to WHO a health system includes “*all the activities whose primary purpose is to promote, restore, and maintain health*,” which encompasses all organizational and individual efforts that impact health, beyond “*the pyramid of publicly owned facilities that deliver personal health services*” (1). This definition tries to capture the wide-ranging structure and function of the health system—an aspect that other existing definitions fail to consider. Nevertheless, our focus has mainly been on what constitutes the health system, its functions, desired outcomes, and its values (11, 12); therefore, our challenge is to clarify the definitions of multiple levels and domains involved in health systems. In this regard, a comprehensive and integrated framework can help better understand, evaluate, and resolve the current issues in health systems.

In this communication, we present a new framework for defining the health system from a broader perspective.

## The proposed framework

The standard systems approach incorporates an “input” that undergoes a “process” to achieve a specific “output,” which then enables the system to meet its “outcomes” of interest and exert a desired “impact.”

**Figure 1:**
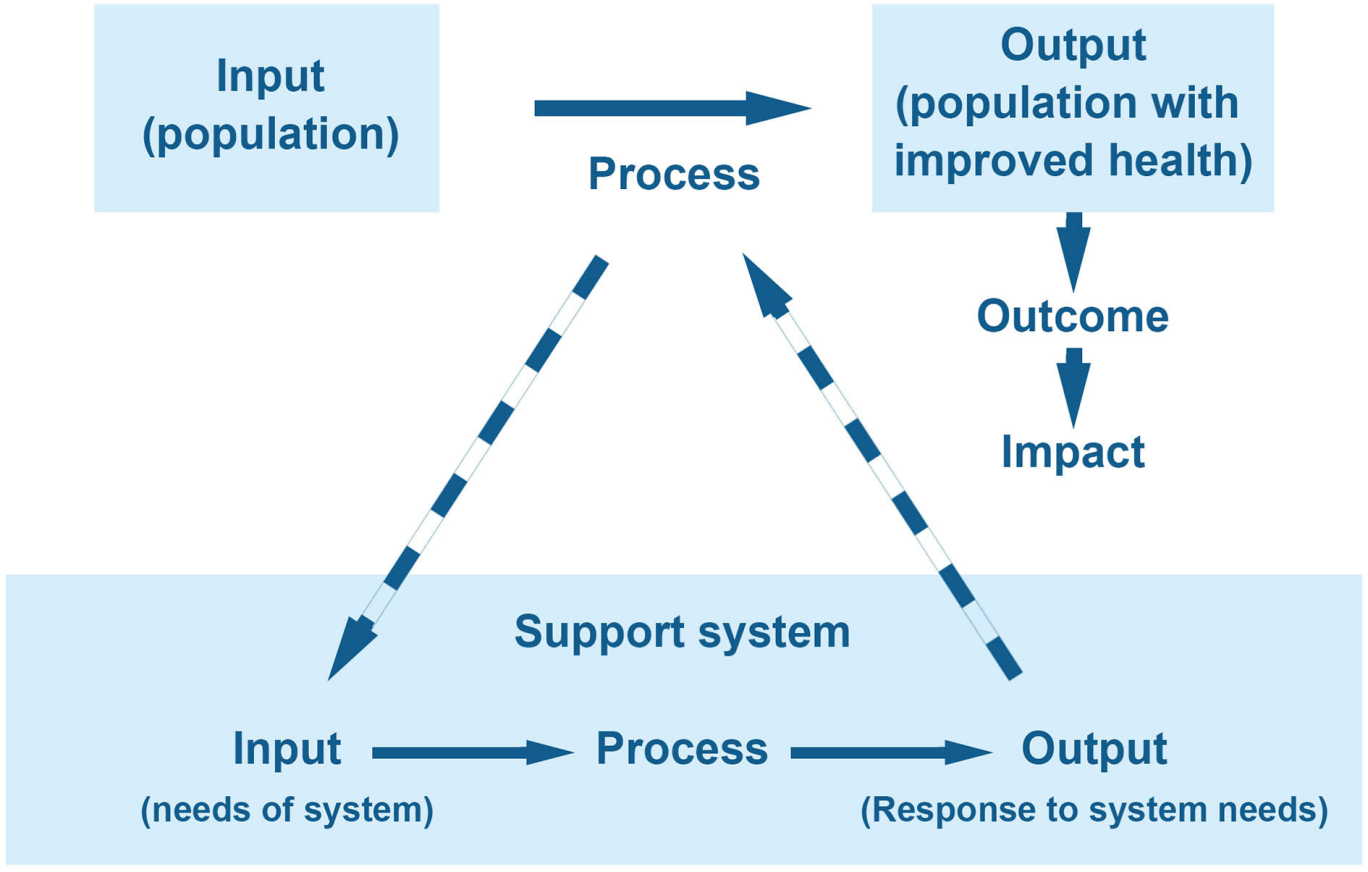
Overview of health system structure.

According to the definition of WHO presented above, the mission of health systems is to improve health in the target population (1, 13). In its simplified form, therefore, the population is the system's input, and the process involves activities aimed to deliver a population with improved health as output (Figure 1).

### Input

Quantification and assessment of inputs is a crucial aspect of a system's evaluation, but defining inputs is elusive. In existing definitions, human resources, healthcare infrastructure, or even funding are often considered inputs (4, 13, 14). But the true input, which is the target of the process and is meant to be modified by the system, is the population and its level of health. The population is often considered as an external beneficiary and a recipient of services (2); however, a systems approach to health should prioritize the population. In this suggested framework, the population takes center stage, as we emphasize that the institutions and individuals who provide health services are not the whole systems, but they are part of the process of performing important functions of the system. The population itself can then be stratified based on health status for better characterization of the input, and each stratum can be perceived as a sub-input.

### Process

**Figure 2:**
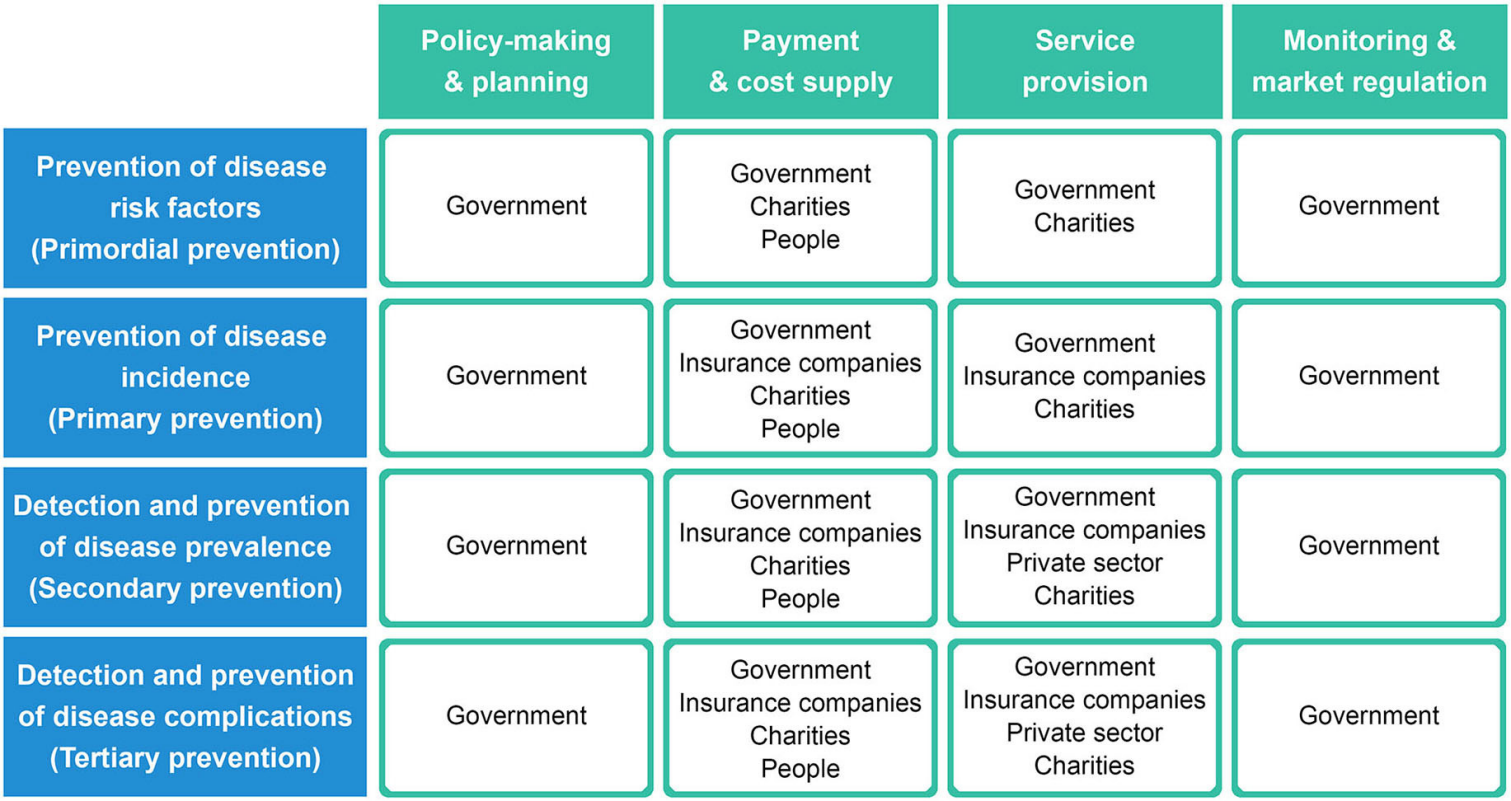
A simplified framework showing how system actors are tasked with different functions of the system process (rows) in each core domain (columns). Each cell represents a subsystem that is assigned to the specific actor(s) responsible.

The collection of efforts, strategies, and structures that are implemented in coordination to improve the health status of the population can be regarded as a process, which includes different functions of the health system, and each function itself can be interpreted and examined in core domains of policy making and planning, resource generation, service provision, monitoring, and regulation (Figure 2).

### System actors

Actors in the system are part of the process and are defined as any individual or organization that provides or receives health services. Government is a prominent actor involved in policymaking and planning, monitoring, and regulating the system. In certain circumstances, the government can be involved in resource generation and service provision to enhance outcomes; however, it is often not clear when and how this should be done, and how to evaluate the cost-effectiveness of this strategy. With the existing ambiguity in health system definition and the inability to compare system parameters between countries, the degree of government involvement in the health system is mostly determined by subjective preferences or national ideologies. Notably, we emphasize all branches of the government and not only the health department or ministry, as the state contributes as an actor through all policies that concern health.

The private sector, insurance companies, charities, and people themselves are other actors in the system. The private sector is distinctively active where there is profit, and it is up to governments to regulate the market to produce incentives for private sector participation. Insurance acts as an important factor to determine health-related costs and facilitates receiving timely interventions. Insurance companies can either belong to the private sector or be government-controlled. Charities are unique in that they do not seek financial gain, yet they can intervene wherever they deem necessary to increase the system's efficiency. People play a role in the system through self-care, good health knowledge, and most importantly the role of receiving care.

Figure 2 demonstrates a simplified approach to how actors engage in different functions of the system process in each core domain. In this framework, each cell in Figure 2 can represent a subsystem that is assigned to the specific actor(s) responsible for that domain, and then the efficiency of each cell can be evaluated with appropriate indicators.

### Output

Quantification and evaluation of the system's output are even more challenging than defining the input (14, 15). Since the raison d'être of the health system is better health in the society (1), the output can be described as the population with improved health outcomes. Indeed, the output can also be stratified into different levels of health status like the input and should be assessed with appropriate output indicators in terms of its attributes. For instance, the output can be the reduction in the number of incident diseases or the number of people who receive education or get vaccinated.

### Outcome and impact

Health outcomes have been extensively examined with a systems approach (7, 15). Outcomes refer to the objectives of activities performed in the health system process, e.g., an outcome of hypertension screening is to reduce the incident cardiovascular events associated with high blood pressure. Ultimately, the desired outcome of the system is the reduction of morbidity and mortality. On the other hand, system impact is concerned with the health status of the target population. The most important impact is of course improved life expectancy and health expectancy, which refers to disability-free and active life in good perceived health (16).

### System environment

Health system is not isolated and is affected by its territorial ecosystem and other social structures. The bilateral relationships of the health system with other social systems such as education, economy, and sources of power determine almost all aspects of health in the society.

### Support systems

Several systems that are commonly regarded as components of a health system can be better defined as support systems. This new definition is helpful in characterizing each component and avoids common problems in analyzing the system and assigning objectives. A support system is defined according to three properties:

A) Its existence takes meaning with the main system. If the main system did not exist, there would be no reason for the support system.
B) Its input is a need of the main system.
C) Its output is used in the process of the main system (Figure 1).

Crucially, this framework serves to simplify the evaluation of these support systems, not undermine their value as accessories. For clarification, two important support systems are discussed herein.

*Health evidence production system (HEPS)* consists of a health research system (HRS) and a health information system (HIS). *HEPS* input is the collection of questions and hypotheses created in Figure 2 cells, and its output is the evidence that will then be used in the health system process.

*Education, development, and support of human resources* should be regarded as a support system and not part of the health system itself. In this regard, the main system's need for trained professionals is the input, and the outputs are professional health care providers who are part of the process in the health system. Notably, this support system should also be assigned the goal of improving the work life of health care workers (17). Notwithstanding this need, the objectives of this support system are sometimes overlooked, and its output is not tailored to the requirements of the health system.

### System values

Values should not be mistaken with objectives, aims, goals, or outcomes. A system cannot function without its values, and its output is not meaningful without meeting system values first. Safety, equity, accountability, international collaboration, quality, and safety are better defined as values of the system. Values act as the inner compass of the system and are there to assure it remains on the right path. Every system has its own set of values, which are decisive in defining goals and directions. While values are instrumental to the success of the system and should be evaluated and monitored, they are distinct from goals.

## Conclusion

In this communication, we propose a new concept of a health system based on the classic attributes of a system. First, we attempt to simplify the description of inputs and outputs of the health system. Establishing the correct definition of inputs and outputs—a feature that has often been neglected—is crucial in health systems, since analyzing and comparing outputs with regards to inputs, or vice versa is the core of efficiency measurement in any system (14, 18). As mentioned, the input and output can be stratified based on health status but can be further characterized by demographics, insurance coverage, perceptions of health, health literacy, and so forth. The properties of the input are among the factors that determine the activities in the process and should be considered when comparing health systems between countries.

Second, we propose using the concept of support systems to separately evaluate various levels and functions of the health system. This approach breaks down parts of the system, which are hard to fit under a single definition, into smaller support systems that are assigned different functions and are easier to evaluate.

Third, we suggest a differentiation between system values, aims, and outcomes. Importantly, each element should be assessed with its specific indicators to avoid confusion in the system.

Based on these concepts, future efforts are needed to improve this health system framework. The next steps may focus on the analysis of sub-systems and support systems and attempt to determine the role of each actor in the system with respect to its capabilities.

## Data availability statement

The original contributions presented in the study are included in the article, further inquiries can be directed to the corresponding author.

## Author contributions

FA and HE contributed to conception and design. FA and HT wrote the draft manuscript. All authors contributed to the article and approved the submitted version.

## Conflict of interest

The authors declare that the research was conducted in the absence of any commercial or financial relationships that could be construed as a potential conflict of interest.

## Publisher's note

All claims expressed in this article are solely those of the authors and do not necessarily represent those of their affiliated organizations, or those of the publisher, the editors and the reviewers. Any product that may be evaluated in this article, or claim that may be made by its manufacturer, is not guaranteed or endorsed by the publisher.

## Abbreviations

WHO: World Health Organization
HEPS: health evidence production system
HRS: health research system
HIS: health information system.

## References

[1] World Health Organization. The World health report : 2000 : Health systems : improving performance. World Health Organization (2000). Available online at: https://apps.who.int/iris/handle/10665/42281 (accessed August 25, 2021).

[2] Frenk J. The global health system: strengthening national health systems as the next step for global progress. PLoS Med. (2010) 7:e1000089. 10.1371/journal.pmed.1000089 20069038 PMC2797599

[3] Frenk J. Strengthening health systems to promote security. Lancet. (2009) 373:2181–2. 10.1016/S0140-6736(09)60002-7 19150131

[4] Murray CJ Frenk J. A framework for assessing the performance of health systems. Bull World Health Organ. (2000) 78:717. 10916909 PMC2560787

[5] Berwick DM Nolan TW Whittington J. The triple aim: care, health, and cost. Health Aff. (2008) 27:759–69. 10.1377/hlthaff.27.3.759 18474969

[6] Agency for Healthcare Research Quality. Defining Health Systems. Content last reviewed September 2017. Rockville, MD: Agency for Healthcare Research and Quality. Available online at: https://www.ahrq.gov/chsp/chsp-reports/resources-for-understanding-health-systems/defining-health-systems.html (accessed August 23, 2021).

[7] Clarkson J Dean J Ward J Komashie A Bashford T. A systems approach to healthcare: from thinking to -practice. Futur Healthc J. (2018) 5:151–5. 10.7861/futurehosp.5-3-151 31098557 PMC6502599

[8] Busse R Blümel M. Germany: health system review. Health Syst Transit. (2014) 16:1–296. 25115137

[9] Meng Q Mills A Wang L Han Q. What can we learn from China's health system reform? BMJ. (2019) 365:l2349. 10.1136/bmj.l2349 31217222 PMC6598719

[10] Ridic G Gleason S Ridic O. Comparisons of health care systems in the United States, Germany and Canada. Mater Socio Medica. (2012) 24:112. 10.5455/msm.2012.24.112-120 23678317 PMC3633404

[11] Mold J. Goal-directed health care: redefining health and health care in the era of value-based care. Cureus. (2017) 9:e1043. 10.7759/cureus.1043 28367382 PMC5360382

[12] Badash I Kleinman NP Barr S Jang J Rahman S Wu BW. Redefining health: the evolution of health ideas from antiquity to the era of value-based care. Cureus. (2017) 9:e1018. 10.7759/cureus.1018 28348937 PMC5346014

[13] World Health Organization. Everybody's business – strengthening health systems to improve health outcomes : WHO's framework for action. Available online at: https://apps.who.int/iris/handle/10665/43918 (accessed August 23, 2021).

[14] Chapman C Kern A Laguecir A Quentin W. Management accounting and efficiency in health services: the foundational role of cost analysis. In: Cylus J, Papanicolas I, Smith PC, editors. Health System Efficiency: How to Make Measurement Matter for Policy and Management. Copenhagen: European Observatory on Health Systems and Policies (2016) (Health Policy Series, No 46). 28783269

[15] Kaplan G Bo-Linn G Carayon P Pronovost P Rouse W Reid P. Bringing a systems approach to health. NAM Perspect. (2013) 3:1–24. 10.31478/201307a

[16] Saito Y Robine J-M Crimmins EM. The methods and materials of health expectancy. Stat J IAOS. (2014) 30:209–23. 10.3233/SJI-140840 30319718 PMC6178833

[17] Bodenheimer T Sinsky C. From triple to quadruple aim: care of the patient requires care of the provider. Ann Fam Med. (2014) 12:573–6. 10.1370/afm.1713 25384822 PMC4226781

[18] Kindig D Stoddart G. What is population health? Am J Public Health. (2003) 93:380–3. 10.2105/AJPH.93.3.380 12604476 PMC1447747

